# Quality of Life of Postoperative Photon versus Proton Radiation Therapy for Oropharynx Cancer

**DOI:** 10.14338/IJPT-18-00032.1

**Published:** 2018-11-30

**Authors:** Sonam Sharma, Olivia Zhou, Reid Thompson, Peter Gabriel, Ara Chalian, Christopher Rassekh, Gregory S. Weinstein, Bert W. O'Malley, Charu Aggarwal, Joshua Bauml, Roger B. Cohen, J. Nicholas Lukens, Samuel Swisher-McClure, Alireza F. Ghiam, Peter H. Ahn, Alexander Lin

**Affiliations:** 1 Department of Radiation Oncology, University of Pennsylvania, Philadelphia, PA, USA; 2 College of Arts and Sciences at the University of Pennsylvania, Philadelphia, PA, USA; 3 Department of Otolaryngology-Head and Neck Surgery, University of Pennsylvania, Philadelphia, PA, USA; 4 Department of Medical Oncology, University of Pennsylvania, Philadelphia, PA, USA

**Keywords:** oropharyngeal cancer, quality of life, proton therapy, intensity-modulated radiation therapy

## Abstract

**Purpose:**

Quality of life (QOL) for patients with oropharyngeal squamous cell cancer is negatively affected by conventional radiation (RT) owing to radiation exposure to normal tissues. Proton therapy, via pencil beam scanning (PBS), can better spare many of these tissues, and may thereby improve QOL.

**Patients and Methods:**

Patient-reported outcomes were prospectively collected from patients treated from April 2013 to April 2015. Patients were treated with PBS or intensity-modulated radiation therapy (IMRT) via volumetric arc therapy after transoral robotic surgery. Validated QOL questionnaires were collected before RT, and 3, 6, and 12 months post RT.

**Results:**

Sixty-four patients were treated with adjuvant RT after transoral robotic surgery, 33 (52%) with volumetric arc therapy, and 31 (48%) with PBS. Both groups were similar in terms of age, site, stage, and dose delivered. Patients receiving PBS had significantly less dose to many normal structures than those receiving IMRT. These dosimetric advantages with PBS were reflected in higher scores in head and neck specific, as well as general, QOL measures. Most notable was significantly less xerostomia with PBS, on multiple patient-reported outcomes at multiple timepoints (6 and 12 months).

**Conclusion:**

Pencil beam scanning, when compared to IMRT, confers a significant dosimetric advantage to many normal organs at risk, with a corresponding benefit in multiple patient-reported QOL parameters in patients receiving adjuvant RT for oropharyngeal squamous cell cancer.

## Introduction

The increasing incidence of oropharyngeal squamous cell carcinoma (OPSCC) in the United States and Europe has been driven largely by increasing rates of oral human papilloma virus (HPV) infection [1]. Given the generally favorable prognosis of patients with HPV-related OPSCC [2], there is an expanding focus on decreasing the toxicity of treatment while preserving efficacy [3].

Curative treatment of OPSCC commonly involves radiation with or without chemotherapy after surgery or in the definitive, nonoperative setting. The clinical benefits of elective treatment of the neck in OPSCC are well established [4], but comprehensive nodal irradiation can lead to significant and sometimes severe short- and long-term toxicities. This is an especially important issue for patients with HPV-positive OPSCC who are relatively young and healthy, often cured of their disease, and will live for decades beyond their treatment. Even the most technically advanced forms of conformal radiation therapy, such as intensity-modulated radiation treatment (IMRT), still cause acute grade-3 side effects in many patients [5]. Acute radiation toxicities in turn can then lead to late toxicities that can negatively impact quality of life (QOL) [6, 7]. Efforts to reduce toxicity and improve patient QOL are therefore of paramount importance.

Proton beam therapy (PBT), with its unique physical beam characteristics (Bragg peak), consistently results in improvements in dosimetry and normal tissue sparing [8–12]. However, translation of dosimetric benefits to improved clinical outcomes has been limited in the setting of head and neck cancers. This study is the first of its kind to report an association between dosimetric advantages and subsequent clinical outcomes in patients receiving postoperative radiation therapy in the treatment of oropharynx cancer, and the first to report late outcomes at 12 months.

## Materials and Methods

### Patients

This is an institutional review board–approved study of 64 patients with OPSCC, treated at the University of Pennsylvania (between 2013 and 2015) initially with transoral robotic surgery and selective neck dissection, followed by adjuvant (n = 64) radiation, with or without chemotherapy (according to standard indications) [13]. Whether patients received PBT or IMRT via volumetric modulated arc therapy (VMAT) was determined solely on the basis of insurance approval for each patient.

### Treatment Planning and Dosimetric Comparisons

At time of treatment planning, normal structures representing organs at risk were contoured for all patients. Contouring followed previously published guidelines [14]. All patients received treatment to the primary site and bilateral neck, with standard, accepted postoperative fractionation and doses of 60 to 66 Gy [15, 16], and with target delineation of primary and secondary echelon nodal regions, based on published and commonly used guidelines [17]. Treatment planning for both PBT and VMAT was performed via Eclipse version 11 (Varian Medical Systems, Palo Alto, California). Proton beam therapy was planned and delivered via a pencil beam scanning (PBS), single-field uniform dose technique. Dosimetric data for all structures were extracted from Eclipse, with dose-volume histogram (DVH) data exported into the R software package version 3.2.1 (R Foundation for Statistical Computing, Auckland, New Zealand).

### Assessment of Patient-Reported QOL Outcomes

Patient-reported QOL questionnaires were prospectively obtained at initial radiation therapy consultation (pretreatment) and at 3, 6, and 12 month follow-up intervals and included the following: European Organization for Research and Therapy of Cancer (EORTC) QLQ-30 version 3 EORTC OLO-H&N35 and the Groningen (GRIX) Xerostomia, Work Status, and Performance Status Scale–Head and Neck Cancer questionnaires. All questionnaires had previously been externally validated [18, 19]. The EORTC questionnaire was used to determine General Health Domain, Physical and Role Function, overall xerostomia, dental issues, head and neck pain, and fatigue scores. The GRIX questionnaire was used to analyze separate components of dry mouth such as differentiating between day and night xerostomia and separate subscales for sticky saliva. As per the EORTC and GRIX guidelines, composite scores examining specific domains were linearly converted to a 0 to 100 scale, with a difference of 10 in the composite scale considered to be clinically significant [20].

### Statistical Analysis

All statistical analyses were completed by using Stata, version 13.1 (StataCorp LP, College Station, Texas). Patient characteristics were compared by using a *t* test for continuous and Fisher exact test for discrete variables. Dosimetric comparisons of salivary structures were performed by using a *t* test with correction for multiple comparisons using the Bonferroni method, ascribing a *P* value of .004 as statistically significant. For patient-reported outcomes (PROs) with continuous variables *t* test was used, while Wilcoxon rank sum test was used for comparison of categorical variables reported on the PROs. For parameters with binary outcomes, Fisher exact test was used. Associations were noted as statistically significant with a 2-sided *P* value <.05.

## Results

Patient and disease characteristics were similar between both groups (PBS vs. IMRT) of patients, including age, sex, stage, nodal status, radiation dose, and receipt of concurrent systemic therapy (**Table 1**).

**Table 1:** Patient characteristics.

	**VMAT (n = 33)**	**PBS (n = 31)**	***P*** **value**
Age, y, mean	58	60	.365
Sex			.561
Male	27 (82%)	27 (87%)	
Female	6 (18%)	4 (13%)	
Primary site			.800
Tonsil	20 (61%)	20 (65%)	
Base of tongue	13 (39%)	11 (35%)	
Stage			.796
I-III	5 (15%)	4 (13%)	
IVA	28 (85%)	27 (87%)	
T stage			.272
Tis, T1, T2	32 (97%)	28 (90%)	
T3	1 (3%)	3 (10%)	
Nodal status			.195
N0	1 (3%)	2 (6%)	
N1-N2b	29 (88%)	29 (94%)	
N2c-N3	3 (9%)	0 (0%)	
Median dose, Gy	62.6	61.7	.288
Chemotherapy			.110
None	19 (58%)	19 (61%)	
Cisplatin	13 (39%)	7 (23%)	
Cetuximab	1 (3%)	5 (16%)	

**Abbreviations**: VMAT, volumetric arc therapy; PBS, pencil beam scanning.

**Figure 1:**
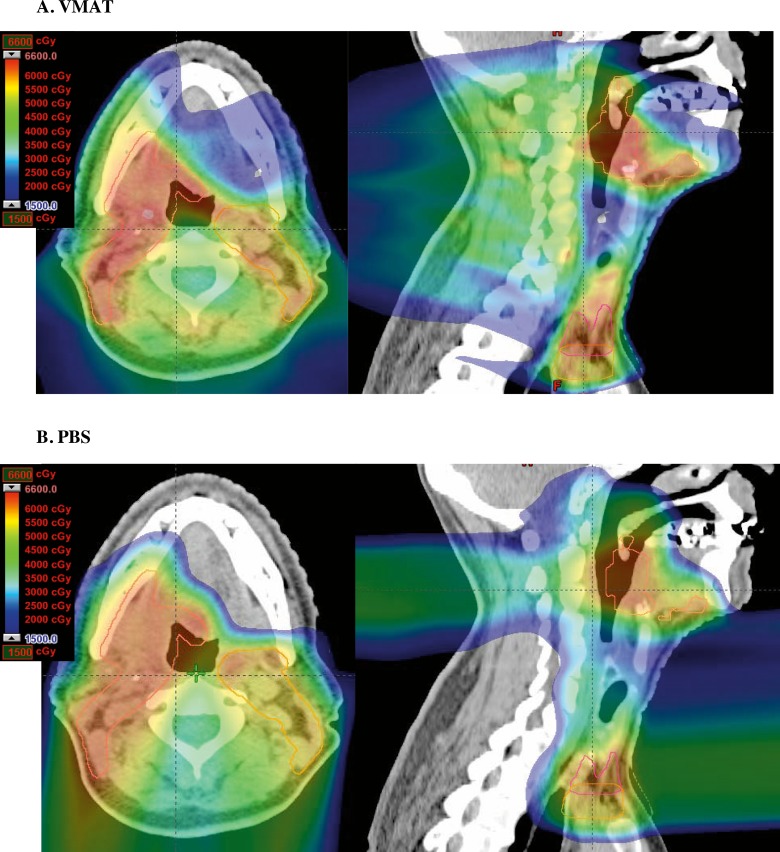
VMAT versus PBS plan comparison: axial (left) and sagittal (right) slices of representative radiation plans for adjuvant radiation therapy in a patient with T1N2aM0 stage IVA base of tongue carcinoma status post transoral robotic surgery and neck dissection, showing VMAT (A) and PBS (B) radiation plans (60 Gy in 30 fractions) for the same patient. The PBS plan demonstrates lower dose to oral cavity structures than VMAT. Abbreviations: PBS, pencil beam scanning; VMAT, volumetric arc therapy.

Representative radiation plans for a patient treated with adjuvant radiation for OPSCC with VMAT and PBS are shown in **Figure 1**, with significant sparing of the anterior oral cavity with PBS. Mean radiation dose was significantly lower for PBS than VMAT patients for all salivary structures other than the ipsilateral parotid and ipsilateral submandibular glands, with the contralateral parotid and submandibular glands, ipsilateral and contralateral buccal mucosa, upper and lower lips, and oral tongue receiving one-third to 10-fold lower mean dose with PBS (**Table 2**). When combining all structures known to produce saliva in the oral cavity and defining this as a separate composite structure, PBS resulted in significantly lower mean dose to the composite oral cavity structure than VMAT (35.1 Gy VMAT vs. 21.2 Gy PBS, *P* < .0001). Both VMAT and PBS patients had a 0% rate of percutaneous endoscopic gastrostomy tube dependence at 6 months.

**Table 2:** Dosimetric comparison of all patients by treatment cohort, representing OARs important for salivary function.

**Patients (n = 64)**	**VMAT (n = 33), Gy**	**PBS (n = 31), Gy**	***P*** **value**
**Mean dose (95% CI)**			
Ipsilateral parotid	32.83 (29.43, 36.32)	35.29 (32.09, 38.48)	.295
Contralateral parotid	20.78 (18.34, 23.22)	11.90 (9.71, 14.09)	**<.0001**^a^
Ipsilateral submandibular	60.11 (59.40, 60.81)	60.52 (59.82, 61.22)	.398
Contralateral submandibular	38.01 (32.58, 43.43)	28.75 (24.12, 33.38)	.0107
Ipsilateral sublingual	42.69 (36.72, 46.04)	32.47 (27.68, 37.26)	.**0009**^a^
Contralateral sublingual	36.03 (32.37, 39.69)	7.54 (4.85, 10.22)	**<**.**0001**^a^
Ipsilateral buccal	25.97 (23.85, 28.09)	9.48 (5.94, 13.02)	**<**.**0001**^a^
Contralateral buccal	18.84 (13.66, 18.10)	1.34 (0.70, 1.99)	**<**.**0001**^a^
Hard palate	15.86 (13.71, 18.00)	5.66 (2.55, 8.77)	**<**.**0001**^a^
Soft palate	36.82 (33.90, 39.72)	30.59 (26.05, 35.13)	.202
Tongue	39.94 (37.45, 42.44)	25.98 (23.04, 28.91)	**<**.**0001**^a^
Upper lip	10.38 (8.81, 11.95)	1.40 (0, 3.09)	**<**.**0001**^a^
Lower lip	20.38 (18.26, 22.50)	2.82 (0.35, 5.29)	**<**.**0001**^a^
Oral cavity	35.11 (33.14, 37.08)	21.23 (18.82, 23.64)	**<**.**0001**^a^

**Abbreviations**: OARs, organs at risk; VMAT, volumetric arc therapy; PBS, pencil beam scanning; CI, confidence interval.

Note: Mean dose with standard error of the mean is represented for these structures.

a Statistically significant results (ascribed at *P* = .0036 using Bonferroni correction for multiple comparisons).

Patients treated with PBS reported significantly less head and neck pain on the EORTC scale than patients treated with VMAT at 12 months (22.0 VMAT vs. 8.3 PBS, *P* = .01; **Table 3**).

**Table 3:** Patient-reported outcomes representing quality-of-life parameters for patients treated with VMAT or PBS for OPSCC.

	**3 months**			**6 months**			**12 months**		
	**VMAT**	**PBS**	***P*** **value**	**VMAT**	**PBS**	***P*** **value**	**VMAT**	**PBS**	***P*** **value**
Fatigue	26.50	26.50	.63	20.47	8.50	.07	22.22	4.86	.17
H&N pain	28.85	25.00	.34	18.86	8.33	.08	21.97	8.33	.**011^a^**
Painkiller use (%)	35.71	30.77	1.00	21.05	16.67	1.00	36.36	17.65	.38
Xerostomia	47.62	50.00	.96	52.63	39.58	.14	54.55	23.53	.**003^a^**
Moderate-severe dry mouth	57.14	50.00	1.00	63.16	22.22	.**02^a^**	50.00	11.76	.**038^a^**
Xerostomia day	43.06	41.20	.81	39.20	25.80	.**038^a^**	33.33	19.61	.06
Xerostomia night	47.01	33.33	.11	35.10	22.80	.**042^a^**	30.56	17.65	.10
Sticky saliva day	38.46	29.37	.38	20.47	15.43	.60	22.22	17.65	.31
Sticky saliva	45.24	48.72	.81	26.32	27.08	.90	39.39	27.45	.38
Dental problems	19.05	0.00	.**016^a^**	17.54	1.96	.**048^a^**	21.21	5.88	.13
Physical function	87.62	88.10	.83	89.47	97.04	.**006^a^**	87.88	96.86	.24
Role function	70.24	80.77	.43	76.32	96.30	.**0008^a^**	78.79	97.92	.**041^a^**
Global health	66.03	69.05	.41	73.15	83.33	.09	72.73	81.86	.13

**Abbreviations**: VMAT, volumetric arc therapy; PBS, pencil beam scanning: OPSCC, oropharyngeal squamous cell cancer; H&N, head and neck.

a Statistically significant results.

To determine whether the improvement in dosimetric endpoints to oral cavity salivary structures correlated with patient-reported xerostomia, we used PROs from the EORTC and GRIX xerostomia scales (higher score indicating worse xerostomia). Pencil beam scanning conferred a significant benefit in general EORTC xerostomia scores at 12-month follow-up (54.6 VMAT vs. 23.5 PBS, *P* = .003; **Table 3**). While PBS patients showed improvement in xerostomia endpoints over time from 3 to 12 months, VMAT patients reported stable or worsening xerostomia outcomes over this period in all cohorts. There was a benefit favoring PBS over VMAT in rates of moderate to severe general xerostomia as reported on the EORTC questionnaire starting at 6 and 12 months (63.2 VMAT vs. 22.2 PBS at 6 months, *P* = .02; 50 VMAT vs. 11.8 PBS at 12 months, *P* = .038; **Table 3**). Similar findings favored PBS over VMAT in a more granular assessment that included daytime and nighttime xerostomia on the GRIX questionnaire, with no significant differences in terms of sticky saliva. In addition to fewer reported problems with xerostomia, patients receiving PBS reported significantly lower rates of posttreatment dental problems than VMAT patients overall (17.5 VMAT vs. 2.0 PBS at 6 months, *P* = .048; **Table 3**).

Global health status scores on the EORTC questionnaire showed improvement (higher score is better) between 3 and 6 months post treatment in those receiving PBS versus IMRT. Patients treated with PBS had a trend toward improved global health status scores at 6 months (73.2 VMAT vs. 83.3 PBS, *P* = .09; **Table 3**). There were also significant differences favoring protons in terms of role function at 6 and 12 months (76.3 VMAT vs. 96.3 PBS at 6 months, *P* = .0008; 78.8 VMAT vs. 97.9 PBS at 12 months, *P* = .041; **Table 3**).

## Discussion

Our study of patients who received adjuvant radiation therapy for OPSCC shows that improved dosimetric sparing of organs at risk with PBS confers an advantage to clinical toxicity outcomes during follow-up of up to 1 year. A previously published study of 81 patients treated for OPSCC suggested a benefit favoring PBS compared to IMRT at about 6 weeks after treatment [21]. Our study contributes to the existing literature, focusing on a homogeneous patient population (postoperative oropharynx cancer requiring adjuvant radiation therapy), and is the first to report patient outcomes at 12 months.

High-quality studies that examine the potential clinical benefit associated with new technologies such as proton therapy are clearly needed. While there is no debate that the physical characteristics of proton radiation can spare organs at risk, published data demonstrating that these dosimetric gains translate to clinical gains are limited. Efforts are underway to formally address this question via a prospective, randomized trial [22]. Results from such studies will not be available for many years. Preliminary information on clinical outcomes and gains associated with proton therapy is important to help current patients, physicians, and payers make the best treatment decisions. Our study fulfills this need by using prospectively collected and well-validated PRO questionnaires over the course of a year post RT.

There are limitations to our study. Although PROs were collected prospectively, our patients were not assigned treatment (PBS vs. IMRT) via a prospective, randomized controlled trial, but rather via insurance approval. Therefore, even though the groups of patients receiving PBS versus IMRT were similar and well balanced, there exists the possibility for unrecognized bias via unmeasured confounders; however, given that both groups were of similar patient characteristics (**Table 1**) and that patients with HPV-positive OPSCC tend to be homogeneous from a sociodemographic standpoint, we believe that our comparison of the groups, while perhaps not optimal, is still valid. We also acknowledge that current randomized trials of proton therapy versus IMRT for this population will better address such concerns. We also recognize that the total number of patients analyzed is fewer than the number that would be evaluated in a multi-center, randomized trial. We have tried to control for this by focusing our analysis on a relatively homogeneous group of patients treated at a single institution after transoral robotic surgery. However, our limited number of patients allows us to only suggest association between dosimetric gains to organs at risk and improved patient outcomes, rather than demonstrate direct correlation. Again, we are confident that current and future prospective efforts (such as those via prospective trials) will be able to better demonstrate such correlations.

In conclusion, we found that the receipt of PBS, when compared to IMRT, yielded not only dosimetric gains, but also gains in toxicity-specific outcomes, extending to a year out from RT. Patient outcomes are key measures to evaluate and support the application and adoption of a new technology. Our results showing improvements in patient-reported QOL are especially relevant in HPV-related head and neck squamous cell carcinoma in which mitigation of long-term toxicity is of utmost importance given the expected high rates of disease control. Ultimately, our study is but one contribution to the call for clinical data to support the broader use of proton therapy. We, and other centers, will continue work in a collaborative manner to generate additional data to answer these critical questions.
